# Bcl-2-dependent synthetic lethal interaction of the IDF-11774 with the V0 subunit C of vacuolar ATPase (ATP6V0C) in colorectal cancer

**DOI:** 10.1038/s41416-018-0289-1

**Published:** 2018-11-13

**Authors:** Bo-Kyung Kim, Soon Woo Nam, Byung Soh Min, Hyun Seung Ban, Soonmyung Paik, Kyeong Lee, Joo-Young Im, Youngjoo Lee, Joon-Tae Park, Seon-Young Kim, Mirang Kim, Hongsub Lee, Misun Won

**Affiliations:** 1 Personalized Genomic Medicine Research Center, KRIBB, Daejeon, 34141 Korea; 2 The Catholic University of Korea, Incheon St Mary’s Hospital, 56 Dongsuro Bupyunggu, Incheon, 06591 Korea; 3 Serverance Biomedical Science Institute, Yonsei University College of Medicine, Seoul, 03722 Korea; 4 Metabolic Regulation Research Center, KRIBB, Daejeon, 34141 Korea; 5 College of Pharmacy, Dongguk University-Seoul, Goyang, 410-820 Korea; 6 Drug Discovery Team, ILDONG Pharmaceutical Co. Ltd., Hwaseong, Hwaseong, 445-811 Korea; 7 Department of Functional Genomics, KRIBB School of Bioscience, Korea University of Science and Technology (UST), 217 Gajeong-ro, Yuseong-gu, Daejeon, Korea, Daejeon, 34113 Korea

**Keywords:** Macroautophagy, Gene regulation, Drug development, Colon cancer

## Abstract

**Background:**

The IDF-11774, a novel clinical candidate for cancer therapy, targets HSP70 and inhibits mitochondrial respiration, resulting in the activation of AMPK and reduction in HIF-1α accumulation.

**Methods:**

To identify genes that have synthetic lethality to IDF-11774, RNA interference screening was conducted, using pooled lentiviruses expressing a short hairpin RNA library.

**Results:**

We identified *ATP6V0C*, encoding the V0 subunit C of lysosomal V-ATPase, knockdown of which induced a synergistic growth-inhibitory effect in HCT116 cells in the presence of IDF-11774. The synthetic lethality of IDF-11774 with *ATP6V0C* possibly correlates with IDF-11774-mediated autolysosome formation. Notably, the synergistic effect of IDF-11774 and the ATP6V0C inhibitor, bafilomycin A1, depended on the PIK3CA genetic status and Bcl-2 expression, which regulates autolysosome formation and apoptosis. Similarly, in an experiment using conditionally reprogramed cells derived from colorectal cancer patients, synergistic growth inhibition was observed in cells with low Bcl-2 expression.

**Conclusions:**

Bcl-2 is a biomarker for the synthetic lethal interaction of IDF-11774 with *ATP6V0C*, which is clinically applicable for the treatment of cancer patients with IDF-11774 or autophagy-inducing anti-cancer drugs.

## Introduction

Synthetic lethality is traditionally defined as a type of genetic interaction in which the simultaneous mutation of two genes leads to cell death, while mutation of only one of them does not.^1,2^ Recently, the concept of synthetic lethality has been extended to include chemical–genetic and chemical–chemical interactions.^1,2^ Information on synthetic lethality contributes to the understanding of gene functions, mechanisms of drug action, and genetic relations, and to the development of therapeutic strategies for patients with cancer. Genome-wide RNA interference (RNAi) screening has been widely applied to identify various synthetic lethal interactions on the basis of mutated genes or anti-cancer drugs in cancer cells.^3–9^ In a screening of a kinome short hairpin RNA (shRNA) library, vemurafenib, a drug used in sensitive *BRAF*-mutant colon cancers, showed synergy with knockdown of the epidermal growth factor receptor gene (*EGFR*), indicating a synthetic lethal interaction of *B-RAF* and *EGFR*.^6^ The gene encoding neurofibromin, a negative regulator of RAS signaling, confers resistance to tamoxifen based on a genome-wide RNAi screen.^9^ Moreover, a KEAP1-dependent synthetic lethal interaction of *TXNRD1* with AKT inhibitors in cancer has been demonstrated.^10^

Colorectal cancer is one of the most common causes of cancer-related mortality worldwide.^11,12^ According to a report of The Cancer Genome Atlas Project (TCGA) on human colorectal carcinoma, the most frequently mutated genes are *APC*, *TP53*, *KRAS*, *PIK3CA*, *FBXW7*, *SMAD4*, *TCF7L2*, and *NRAS* in non-hypermutated tumors; and *BRAF*, *ACVR2A*, *APC*, *TGFBR2*, *MSH3*, *MSH6*, *SLC9A9*, and *TCF7L2* in hypermutated tumors.^13^ Genetic alterations in the phosphatidylinositide-3-kinase (PI3K) and RAS–MAPK pathways are common, and co-occurrence of alterations in both pathways is observed in approximately one-third of colorectal cancers. In addition, coexistent mutations in exons 9 and 20 of *PIK3CA*, which encodes the p110α catalytic subunit of PI3K, are associated with poor prognosis.^14^ However, the prognostic significance of *PIK3CA* mutations in colorectal cancer remains unclear.

The multikinase inhibitor regorafenib as well as the antibodies bevacizumab and cetuximab, have been approved by the FDA for the targeted therapy of colorectal cancer. However, cytotoxic chemotherapy using 5-fluorouracil (5-FU), oxaliplatin, and irinotecan is used frequently to slow down the growth of incurable metastatic colorectal cancers.^15,16^ Recently, we reported that IDF-11774, a novel clinical candidate, stimulates hypoxia-inducible factor alpha (HIF-1α) degradation, presumably by inhibiting HSP70 chaperone activity.^17,18^ IDF-11774 regulates cancer metabolism by activating AMP-activated protein kinase (AMPK).^17^

To identify genes that are synthetic lethal to IDF-11774, we carried out RNAi screening using pooled lentiviruses expressing an shRNA library and then isolated the genes whose knockdown induced a synergistic growth-inhibitory effect in cancer cells in the presence of IDF-11774. We revealed that *ATP6V0C*, encoding the V0 subunit C of lysosomal V-ATPase, is synthetic lethal with IDF-11774, and this effect correlates with *PIK3CA* mutation and low B-Cell CLL/Lymphoma 2 (Bcl-2) expression. In this study, we provided a rationale for combined treatment with IDF-11774 and an ATP6V0C inhibitor for patients with colorectal cancer that harbor *PIK3CA* mutations and thus, exhibit low Bcl-2 expression.

## Materials and methods

### Chemicals, antibodies, and reagents

Bafilomycin A1 (BM), concanamycin A (CCM), chloroquine (CQ), 3-methyladenine (3-MA), 5-fluorouracil (5-FU), and 2-phenylethynesulfonamide (PES) were purchased from Sigma-Aldrich (St. Louis, MO, USA). siRNAs were obtained from Bioneer (Deajeon, Korea). The following antibodies were used: Bcl-2 (ab32124, Abcam, Cambrige, MA, USA), Bcl-xl (2764, Cell Signaling Technology), Bak1 (3814, Cell Signaling Technology), ATP6V0C (ab104374, Abcam), LC3B (2775, Cell Signaling Technology, Beverly, MA, USA), PARP-1 (9542, Cell Signaling Technology), PIK3CA (4249, Cell Signaling Technology), HA (2367, Cell Signaling Technology), Myc (sc-789, Santa Cruz Biotechnology) and GAPDH (LF-PA0212, AbFrontier Co., Ltd, Seoul, Korea).

### Cell culture and IncuCyte system

The HT29, WiDr, colo320, colo205, SW620, SW480, HCT15, DLD-1, HCC2998, and LoVo human colorectal adenocarcinoma cells were cultured in RPMI-1640 medium containing 10% fetal bovine serum (FBS). The HCT116 human colorectal adenocarcinoma cells and CCD-18Co human colon fibroblast cells were cultured in DMEM medium containing 10% (v/v) FBS. All cells were cultured at 37 °C with 5% CO_2_.

To analyze cell proliferation, the proliferation rates based on cell confluency were determined by live cell imaging (IncuCyte ZOOM system, Essen Bioscience, Ann Arbor, MI, USA). To analyze apoptosis, kinetic caspase-3/7 measurements were assayed using the CellPlayer reagent (Essen Bioscience) as described previously.^19^ The frames of the cells incubating in 96-well plates were captured at 2 h intervals from four separate regions per well using a ×10 objective lens. Cultures were maintained in a 37 °C incubator.

### Establishment and culture of conditionally reprogrammed colorectal cancer cells

Conditionally reprogrammed colorectal cancer cells were established at Yonsei University as previously described.^20^ In brief, a fresh colorectal cancer surgical specimen was washed with PBS and cut into 0.5 mm-sized pieces and then incubated with 0.1% trypsin-EDTA with 5% penicillin-streptomycin at 37 °C for 5 min. The trypsin was neutralized by Dulbecco’s modified Eagle’s medium (DMEM). After centrifugation, the cell pellet was resuspended and incubated in DMEM with collagenase 1A and 5% penicillin-streptomycin at 37 °C for ~1.5 h. The suspended medium was filtered with a cell strainer (70 μm pore size) and then centrifuged. The pellet was resuspended in DMEM and plated on cell culture dishes in F-medium with ROCK inhibitor (Y-27632, STEMCELL Technologies Inc., Seoul, Korea). Irradiated 3T3-J2 cells (generously provided by Dr. Richard Schiegel at Georgetown University) were used as feeder cells. Cells were co-cultured with irradiated 3T3 fibroblasts in F-media (F-12/DMEM medium, 3:1 v/v), 5% FBS, 0.4 μg/ml hydrocortisone, 5 μg/ml insulin, 8.4 ng/ml cholera toxin, 10 ng/ml epidermal growth factor, 24 μg/ml adenine, and 5 µM Y-27632 as described.^21^

### RNAi screening using shRNA expressing lentivirus library

The human module 1 lentiviral shRNA library containing 27,500 shRNAs targeting 5043 genes (Cellecta, Mountain View, CA, USA) was used for synthetic lethal screening according to the manufacturer’s protocol. Briefly, the shRNA library was packaged into lentivirus via HEK293T cells using Lipofectamine and Plus Reagent (Invitrogen, Carlsbad, CA, USA), and then were transduced into HCT116 cells with multiplicity of infection of 0.7 and average 500 cells per shRNA construct. The transfection efficiency was estimated by measuring red fluorescent protein (RFP) by fluorescence activated cell sorting. Then, 48 h post-transduction, HCT116 cells were selected with puromycin (1 μg/ml) for 72 h. An aliquot of the transduced HCT116 cells was preserved as a reference sample (C1). The remaining cells were divided into two samples: one treated with 5 μM IDF-11774 for 96 h and the other with dimethyl sulfoxide (C2). The shRNA insert barcodes were amplified from genomic DNA using a Pfu PCR premix (Bioneer, Daejeon, Korea), and then sequenced using Illumina Hi-Seq 2500 system (Illumina, Inc. San Diego, CA, USA).

### Gene knockdown using siRNA

Gene knockdown was performed by introducing siRNA of the target gene via Lipofectamine 2000 (Invitrogen, Carlsbad, CA, USA) following the manufacturer’s instructions. siRNA sequences are listed in Supplementary Table 1.

### Western blot analysis

Western blot was performed as described.^22^ Cell lysates were separated using sodium dodecyl sulfate-polyacrylamide gel electrophoresis (SDS-PAGE) and then transferred to polyvinylidene fluoride (PVDF) membranes. Western blot bands were quantified using Image J software.

### Reverse-transcription polymerase chain reaction (RT-PCR)

Total RNA was isolated using Trizol reagent (Invitrogen) and cDNA was synthesized using TOPscript™ RT DryMIX (Enzynomics, Seoul, Korea). The sequences of the primers that were used are provided in Supplementary Table 2. PCR products were analyzed by agarose-gel electrophoresis and visualized by ethidium bromide staining.

### Sulforhodamine B (SRB) assay and Combination Index

Growth inhibition of cells was assessed using SRB. The cells fixed with 10% formalin (Sigma-Aldrich) were stained with 0.4% sulforhodamine B. Protein-bound dye was dissolved in 10 mM Tris, and its optical density was measured at 540 nm. Combination Index (CI) values were calculated using the median effect analysis method.^23^ The CI is a quantitative measure of the degree of interaction between two drugs. A synergistic effect is defined as CI < 1, an additive effect as CI = 1, and an antagonistic effect as CI > 1.

### LysoTracker labeling

To stain acidic autolysosomes,^24,25^ the cells were incubated with LysoTracker (Molecular Probes, Life Technologies, Carlsbad, CA, USA) for 30 min at 37 °C. The LysoTracker-labeled cells were observed with a confocal fluorescence microscope (LSM5 Live DuoScan; Carl Zeiss, Stuttgart, Germany) and analyzed using live cell imaging. The number of LysoTracker positive cells was normalized to the number of total cells.

### FITC-Annexin V/PI double staining

An FITC-Annexin V/propidium iodine (PI) double-staining analysis was performed according to the manufacturer’s protocol (BD Biosciences). The cells were treated with IDF-11774 and bafilomycin A1 and then washed twice with pre-chilled phosphate-buffered saline (PBS). The treated cells were stained with FITC-Annexin V staining buffer and PI solution for 15 min at room temperature and then analyzed with a FACSCalibur Flow Cytometer (BD Biosciences).

### Immunofluorescence staining

Immunofluorescence staining was performed as described previously.^19^ The cells were incubated with the LC3B antibody overnight at 4 °C and incubated with conjugated secondary antibody for 1 h at room temperature. The cells were washed with PBS and then stained with 4’,6-diamidino-2-phenylindole for 5 min, and then were observed with a confocal fluorescence microscope (LSM5 Live DuoScan; Carl Zeiss, Stuttgart, Germany). For mRFP-GFP-tagged LC3 assay, cells were transfected with ptfLC3 (Addgene, Cambridge, MA, USA), which contains a gene encoding mammalian expression of rat LC3 fused to mRFP and EGFP, and then observed using confocal fluorescence microscope. Autophgic flux was analyzed by monitoring the change of mRFP-GFP-LC3B fluorescence.

### Immunohistochemistry

The tissue array block of human colorectal cancer and normal tissues were supplied by US Biomax (Rockville, MD, USA). The immunohistochemistry was performed as previously described.^26^ The slides were incubated with the anti-ATP6V0C antibody, respectively, followed by incubation with biotinylated anti-rabbit IgG secondary antibody (Vector Laboratories, Burlingame, CA, USA) and avidin–biotin peroxidase (Vector Laboratories) and visualized using diaminobenzidine tetrahydrochloride (Vector Laboratories). Nuclear counterstaining was accomplished with hematoxylin.

### Immunoprecipitation

Immunoprecipitation assays were performed using Protein A/G agarose beads as recommended by the manufacturer (Santa Cruz Biotechnology, Santa Cruz, CA, USA). HCT116 cells transfected with pCMV3-HA-Bcl-2 and pCMV3-Myc-Bax were treated with 10 µM IDF-11774 and 1 nM bafilomycin A1. Cell extracts (300 µg) were incubated with anti-HA-Agarose at 4 °C overnight. Normal IgG was used as a control. The agarose beads were precipitated and washed. Proteins bound to the beads were detected by western blot analysis.

### In vivo xenograft assay

The in vivo anti-tumor activity of IDF-11774 was analyzed using the human colon cancer HCT116 cells. Cells were injected subcutaneously into 6-week-old female Balb/c nude mice to generate tumors (5 mice per group). After 7 days, the transplanted mice was treated with IDF-11774 (20 mg/kg/day) by oral administration (per os; p.o.) for 15 days. For combination treatment, the mice were injected intraperitoneally (i.p.) with bafilomycin A1 (0.5 mg/kg) daily. Tumor volume (V) was determined using the following equation: V (mm^3^) = (Length × Width × Height) × 0.5.

### Statistical analyses

A Student’s *t-*test or Chi-square test was used for statistical analyses. Bars indicate S.D. and asterisks denote significant differences (***p ≤ 0.005, **p ≤ 0.01, *p ≤ 0.05) of means between the two groups.

## Results

### *ATP6V0C* is identified as a synthetic lethal partner of IDF-11774 by shRNA screening in HCT116 cells

**Fig. 1:**
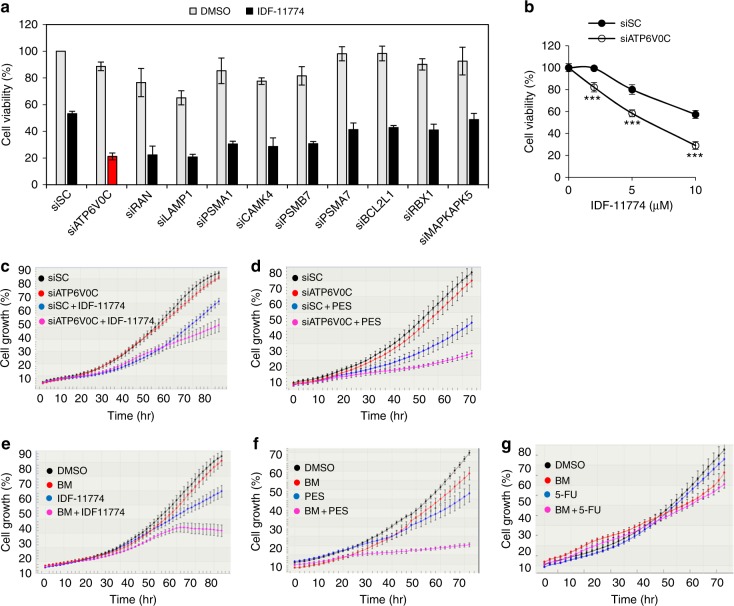
RNA interference (RNAi) screening to identify candidate genes sensitizing HCT116 cells to IDF-11774. **a** Knockdown effect of candidate genes on cell growth of HCT116 cells treated with 10 µM IDF-11774. **b** Effect of *ATP6V0C* knockdown on the growth of HCT116 cells treated with different concentrations of IDF-11774 for 72 h. Cell viability was analyzed by the sulforhodamine B (SRB) assay. Results are shown as the mean ± S.D. of the three independent experiments. **c**, **d** Effect of *ATP6V0C* knockdown on the growth of HCT116 cells treated with 5 μM IDF-11774 or 5 μM 2-phenylethynesulfonamide (PES). **e**–**g** Effect of bafilomycin A1 (BM, 1 nM) on the growth of HCT116 cells treated with 5 μM IDF-11774, 5 μM PES or 5 μM fluorouracil (5-FU). Cell growth was evaluated by live-cell imaging (IncuCyte ZOOM system). Data are presented as the single representative result of three experiments as the mean ± S.D. In each experiment, each treatment was repeated in triplet wells, and each well was analyzed in 4 frames

We performed chemogenetic RNAi screening using pooled lentiviruses expressing a shRNA library in HCT116 cells. Barcode read count analysis revealed 51 candidates whose knockdown induced significant cell death in combination with IDF-11774 treatment as compared with the initial culture (C1) and continued culture without IDF-11774 treatment (C2) as controls, respectively. We analyzed the 51 genes using Search Tool for the Retrieval of Interacting Genes/Proteins (STRING), and identified cell cycle, proteolysis, and lysosome as major gene association networks (Supplementary Figure 1A). The Kyoto Encyclopedia of Genes and Genomes pathway analysis identified proteasome, PI3K-Akt signaling, cell cycle, metabolic pathway, lysosome, and AMPK signaling pathways (Supplementary Figure 1B). As potential genes for synthetic lethality with IDF-11774 in HCT116, we selected 10 genes *ATP6V0C*, *LAMP1*, *BCL2L1*, *RAN*, *RBX1, CAMK4*, *MAPKAPK5, PSMA1*, PSMA7, and *PSMB7* based on pathway analysis and investigated the effect of their knockdown on growth inhibition of cells in the presence of IDF-11774 (Fig. 1a, Supplementary Figure 1C). We observed that knockdown of *ATP6V0C* resulted in substantial growth inhibition in the presence of IDF-11774 (Fig. 1a). We confirmed the dose-dependent chemosensitization of HCT116 cells to IDF-11774 by *ATP6V0C* knockdown (Fig. 1b). Live cell image analysis also indicated that *ATP6V0C* knockdown caused synergistic growth inhibition of HCT116 cells in the presence of IDF-11774 (Fig. 1c). Because IDF-11774 targets HSP70,^18^ we examined the effect of *ATP6V0C* knockdown in the presence of the HSP70 inhibitor 2-phenylethynesulfonamide (PES). As expected, *ATP6V0C* knockdown and simultaneous treatment with PES caused synergistic growth inhibition of HCT116 cells (Fig. 1d). We next confirmed that bafilomycin A1, an ATP6V0C inhibitor, substantially inhibited the growth of cancer cells in combined treatment with IDF-11774 (Fig. 1e). Moreover, combined treatment with bafilomycin A1 and PES also synergistically inhibited HCT116 cell growth (Fig. 1f). However, bafilomycin A1 did not sensitize HCT116 cells to 5-FU, another chemotherapy drug (Fig. 1g).

### *ATP6V0C* knockdown blocks IDF-11774-induced autophagy

**Fig. 2:**
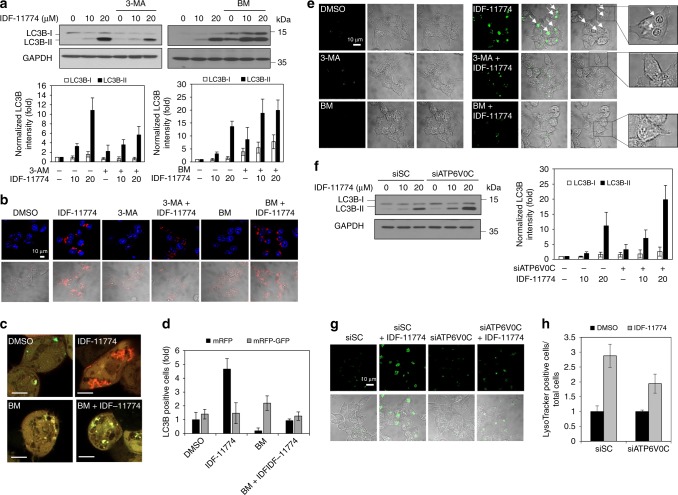
Synthetic lethal interaction of IDF-11774 with *ATP6V0C* in HCT116 cells. **a**, **b** Effect of autophagy inhibitor on LC3B accumulation in cells treated with IDF-11774 as analyzed by western blot analysis (**a**) and immunofluorescence (**b**). HCT116 cells were pre-incubated with 5 mM 3-methyladenine (3-MA) or 1 nM bafilomycin A1 (BM) for 3 h and then treated with IDF-11774 for 18 h. Scale bar, 10 µm. Western blot band intensities were quantified using ImageJ. The signal intensity of LC3B was normalized to that of GAPDH. **c**, **d** Autolysosome formation of cells treated with IDF-11774 and 1 nM bafilomycin A1 (BM). Representative images of confocal fluorescence microscopy (**c**) and quantification of positive cells for mRFP (red) and mRFP-GFP (yellow) in HCT116 cells transfected with ptfLC3 (**d**). Positive cells were counted using fluorescence microscopy (n = ~70). Scale bar, 5 µm. **e** Effect of autophagy inhibitor on autolysosome formation in cells treated with 5 μM IDF-11774 and/or 1 nM bafilomycine A1 for 18 h. Autolysosome formation was assessed by LysoTracker staining. Arrows indicate autolysosomes. Scale bar, 10 µm. **f** Effect of *ATP6V0C* knockdown using siATP6V0C on LC3B accumulation in cells treated with 5 μM IDF-11774, as analyzed by western blotting. **g**, **h** Effect of *ATP6V0C* knockdown on autolysosome formation, as analyzed by LysoTracker staining (**g**) and the ratio of LysoTracker-positive to total HCT116 cells (**h**). Scale bar, 10 µm

IDF-11774 activates AMPK and inhibits the mTOR pathway,^17^ indicating it induces autophagy. We observed that HCT116 cells treated with IDF-11774 accumulated microtubule-associated protein 1 light chain 3 beta (LC3B)-II, indicating autophagosome formation (Fig. 2a). ATP6V0C located in the lysosomal membrane transports protons to acidify lysosomes for autolysosome formation via autophagosome–lysosome fusion.^27,28^ We examined the effects of 3-methyladenine (3-MA), a PI3K type III inhibitor that blocks autophagosome formation, and bafilomycin A1, which blocks autophagosome–lysosome fusion, on autophagic flux in the presence of IDF-11774.^28,29^ We found that 3-MA suppressed the LC3B I–II conversion and thus reduced LC3B-II accumulation in the presence of IDF-11774 (Fig. 2a). By contrast, bafilomycin A1 enhanced LC3B-II accumulation in HCT116 cells treated with IDF-11774. Immunofluorescence staining confirmed that IDF-11774-induced LC3B-II accumulation was suppressed by pre-treatment with 3-MA, but not by bafilomycin A1 treatment (Fig. 2b). We next examined autolysosome formation using a double-tagged mRFP and GFP-LC3B reporter construct (ptfLC3) to discriminate autophagosomes and autolysosomes;^30,31^ autophagosomes, which were labeled with both mRFP and GFP, emitted yellow fluorescence, whereas autolysosomes emitted red fluorescence because of the degradation of GFP-LC3 in the acidic lysosomal condition. Red fluorescent vesicles increased in cells treated with IDF-11774, indicating the formation of functional autolysosomes by fusion of autophagosomes and lysosomes (Fig. 2c, d). However, red fluorescent vesicles were hardly observed in cells treated with bafilomycine A1 in the presence of IDF-11774, indicating that functional autolysosomes were not formed. Next, we assessed whether functional autolysosome formation can be visualized using LysoTracker, which stains functional, acidified autolysosome.^24,25^ We observed the green fluorescence of LysoTracker mainly around functional lysosomes formed in the presence of IDF-11774 (Fig. 2e). As expected, the green fluorescence of functional lysosomes induced by IDF-11774 was reduced by pre-treatment with 3-MA or bafilomycin A1, indicating that the autophagy inhibitors disrupted IDF-11774-mediated autolysosome formation.

We then determined the effect of *ATP6V0C* knockdown on autophagy in HCT116 cells. *ATP6V0C* knockdown enhanced the LC3B-II accumulation induced by IDF-11774, as shown by western blotting (Fig. 2f), indicating autophagosome accumulation. We used LysoTracker to examine whether the combination of IDF-11774 treatment and *ATP6V0C* knockdown would affect functional autolysosome formation. *ATP6V0C* knockdown decreased IDF-11774-induced green fluorescence, indicating suppression of functional autolysosome formation (Fig. 2g, h). Thus, the inhibition of ATP6V0C blocked IDF-11774-induced functional autolysosome formation, resulting in autophagosome accumulation in HCT116 cells.

### Synergistic effect of IDF-11774 and *ATP6V0C* knockdown correlates with IDF-11774-mediated autolysosome formation

**Fig. 3:**
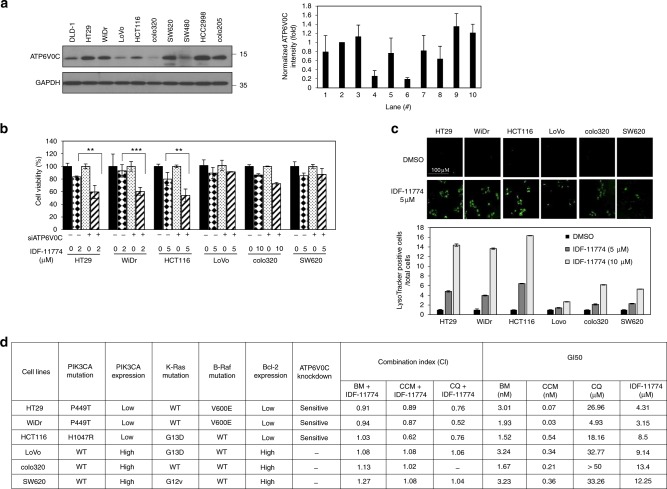
Synergistic effect of *ATP6V0C* and IDF-11774 on the growth of various colorectal cancer cells. **a** Western blot analysis of ATP6V0C levels in human colorectal cancer cells. The signal intensity of ATP6V0C was normalized to that of GAPDH. **b** Effect of *ATP6V0C* knockdown on the sensitivity of human colorectal cancer cells to IDF-11774. Cell viability was analyzed using the sulforhodamine B (SRB) assay. Results are shown as the mean ± S.D. of three independent experiments. **c** LysoTracker staining for autolysosomes in various colorectal cancer cells. LysoTracker-positive cells as observed by fluorescence microscopy. Ratios of LysoTracker-positive to total cells are shown in the graph. Scale bar, 100 µm. **d** Correlation of mutation status or expression of genes and combination index of two compounds. BM (bafilomycin A1), CCM (concanamycin A), and CQ (chloroquine) were used

To understand the clinical significance of ATP6V0C expression in colorectal cancer, we used a colorectal cancer tissue array for immunohistochemical analysis (Supplementary Figure 2). ATP6V0C was highly expressed in 48% of colorectal cancer tissues (*p* *=* 0.0006) and was significantly associated with lymph node metastasis (*p* *=* 0.0002) (Supplementary Figure 2B), suggesting its role in colorectal cancer progression. ATP6V0C expression was variable and high in HT29 and WiDr cells, but low in colo320 cells (Fig. 3a). We first examined whether the ATP6V0C expression level correlated directly with sensitivity to IDF-11774 in various colorectal cancer cell lines. *ATP6V0C* knockdown showed a synergistic effect on growth inhibition by IDF-11774 in WiDr, HT29, and HCT116 cells, but not in LoVo, colo320, and SW620 cells, suggesting that the ATP6V0C expression level was not relevant to IDF-11774 sensitivity (Fig. 3b, Supplementary Table 3). Therefore, we next examined the effect of IDF-11774 on autolysosome formation using LysoTracker. Strong green fluorescence was observed in HT29, WiDr, and HCT116 cells, but not in SW620, colo320, and Lovo cells, upon treatment with IDF-11774 (Fig. 3c). This result suggested that the synergistic cancer cell growth-inhibitory effect of IDF-11774 and *ATP6V0C* knockdown correlates with the ability for autolysosome formation in the presence of IDF-11774.

### *PIK3CA* genetic status is associated with the synthetic lethal interaction of IDF-11774 and functional autolysosome formation

The IDF-11774-bafilomycin A1 combined treatment induced different responses in various colorectal cancer cell lines; therefore, we conducted isobologram analysis to characterize the relationship between IDF-11774 and the ATP6V0C inhibitors, bafilomycin A1 and concanamycin A, or the autophagosome–lysosome fusion inhibitor, chloroquine, to identify genetic marker(s) in the cell lines (Fig. 3d). The ATP6V0C inhibitors demonstrated a synergistic effect (combination index [CI] < 1) with IDF-11774 in HCT116, WiDr, and HT29 cells. However, in LoVo, colo320, and SW620 cells, all three inhibitors showed an antagonistic effect (CI > 1) with IDF-11774, suggesting a crucial role for functional autolysosome formation in the synergistic effect. Therefore, we investigated the relevance of genetic mutations to the CI of IDF-11774 and functional autolysosome inhibition in the cancer cell lines. We found that *PIK3CA* mutation rendered colorectal cells significantly sensitive to the combined treatment with IDF-11774 and inhibitors of autophagosome–lysosome fusion (Fig. 3d). This result suggested that the *PIK3CA* genetic status is probably associated with the synthetic lethality of IDF-11774 and the autophagy inhibitors.

### Combined treatment of IDF-11774 with bafilomycin A1 induces apoptosis in cancer cells

**Fig. 4:**
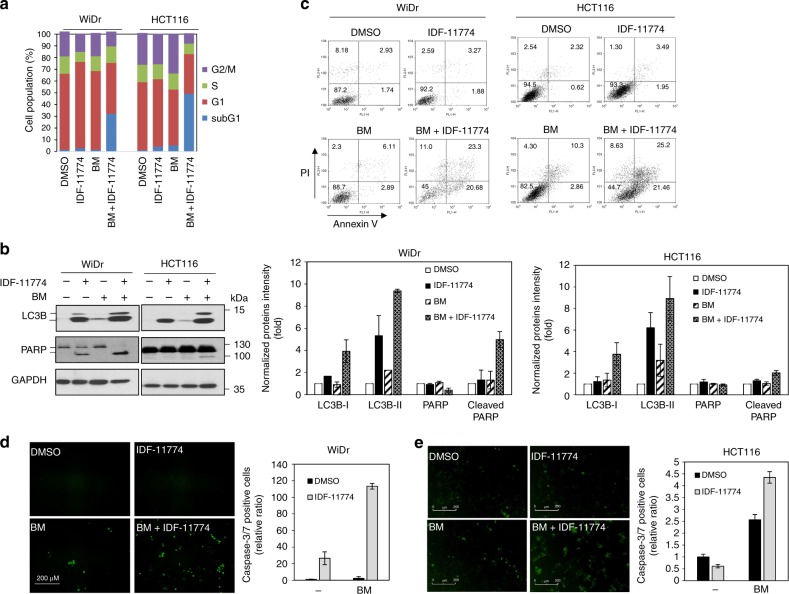
Combined treatment of bafilomycin A1 and IDF-11774 induces apoptosis in colorectal cancer cells. **a** FACScan analysis of cells treated with IDF-11774 and 1 nM bafilomycin A1 for 72 h by propidium iodide (PI) staining. **b** Western blot analysis of cells treated with IDF-11774 and 1 nM bafilomycin A1 for 72 h. The signal intensity of each protein was normalized to that of GAPDH. **c** Scatter-grams of fluorescein isothiocyanate (FITC)-Annexin V/propidium iodide (PI) staining of WiDr and HCT116 cells treated with IDF-11774 and 1 nM bafilomycin A1 for 72 h. **d**, **e** Measurement of caspase-3/7 activity using the CellPlayer kinetic caspase-3/7 reagent. Caspase-3/7-positive cells appeared green under a fluorescence microscope. WiDr (**d**) and HCT116 cells (**e**) treated with IDF-11774 and 1 nM bafilomycin A1 for 72 h using the IncuCyte ZOOM system. Scale bar, 200 µm. The concentration of IDF-11774 was 5 μM in WiDr and 10 μM in HCT116 cells

To clarify the cell death induced by combined treatment with IDF-11774 and bafilomycin A1, we performed flow-cytometric analysis. We found that the G1 cell population of HCT116 and WiDr cells increased in the presence of IDF-11774. However, simultaneous treatment with IDF-11774 and bafilomycin A1 dramatically increased the sub-G1 population of HCT116 and WiDr cells, indicating the occurrence of apoptosis (Fig. 4a). Western blot analysis of cells treated with both IDF-11774 and bafilomycin A1 revealed the accumulation of LC3B-II and cleaved PARP-1, indicating the occurrence of apoptosis (Fig. 4b). Moreover, Annexin V-propidium iodide double staining demonstrated that the combined treatment increased the pro-apoptotic and late-apoptotic populations in both cell lines (Fig. 4c). In addition, caspase-3/7 assays using CellPlayer showed that combined treatment with IDF-11774 and bafilomycin A1 caused a substantial increase in caspase-3/7 activity compared with treatment with IDF-11774 alone in both WiDr (Fig. 4d) and HCT116 (Fig. 4e) cells. These results suggested that IDF-11774 and bafilomycin A1 synergistically inhibited the growth of both WiDr and HCT116 cells by inducing apoptosis through blocking autolysosome formation.

### Bcl-2 plays a crucial role in the synthetic lethality of IDF-11774 and ATP6V0C

**Fig. 5:**
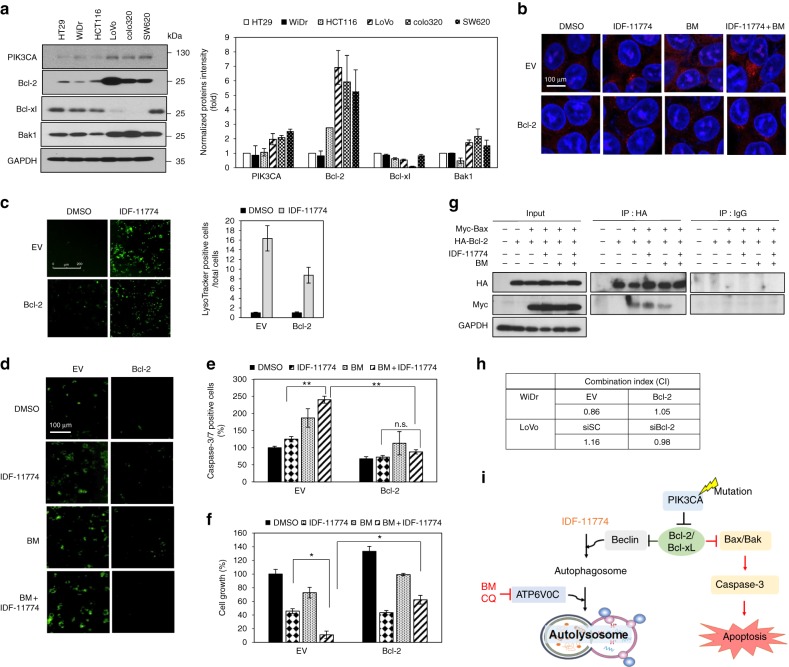
Bcl-2 expression level is crucial for the synthetic lethality of IDF-11774 and bafilomycin A1. **a** Western blot analysis of Bcl-2 family protein levels in human colorectal cancer cells. The signal intensity of each protein was normalized to that of GAPDH. **b**–**f** HCT116 cells transfected with pCMV-SPORT6- *BCL2* were treated with 10 µM IDF-11774 and 1 nM bafilomycin A1. **b** Immunofluorescence of LC3B. Scale bar, 10 µm. **c** Autolysosome formation. Caspase-3/7 activity (**d**, **e**) and cell growth (**f**) were measured by IncuCyte ZOOM system. Graphs are shown as the mean ± S.D. in different regions (n = 4) of the well. **g** Evaluation of the change in the interaction between Bcl-2 and Bax induced by bafilomycin A1 using immunoprecipitation assay. HCT116 cells transfected with pCMV3-HA-*BCL2* and pCMV3-Myc-*BAX* were treated with 10 µM IDF-11774 and 1 nM bafilomycin A1. **h** Combination index of IDF-11774 with bafilomycin A1 in WiDr cells transfected with pCMV-SPORT6-*BCL2* and in LoVo cells transfected with si*BCL2*. **i** Model depicting Bcl-2-dependent synthetic lethal interaction of IDF-11774 and ATP6V0C

When we examined the levels of several proteins involved in PI3K signaling, Bcl-2 was found to be low in both HT29 and WiDr cells bearing the P445T mutation, and in HCT116 cells bearing the H1047R mutation in *PIK3CA*, but high in LoVo, colo320, and SW620, which harbor wild-type *PIK3CA* (Fig. 5a).

Next, we investigated whether Bcl-2 expression affects autophagy and apoptosis during the combined treatment of HCT116 cells with IDF-11774 and bafilomycin A1. *BCL2* overexpression substantially reduced LC3B-II accumulation by treatment with IDF-11774 and bafilomycin A1, indicating suppression of autophagosome formation (Fig. 5b). As expected, IDF-11774-induced autolysosome formation was reduced in HCT116 cells by *BCL2* overexpression, but increased in LoVo cells by *BCL2* knockdown (Fig. 5c, Supplementary Figure 3A). Moreover, *BCL2* overexpression suppressed apoptosis induction by simultaneous treatment with IDF-11774 and bafilomycin A1, as shown by western blot analysis (Supplementary Figure 3B) and caspase-3/7 activity assay (Fig. 5d, e). Notably, *BCL2* overexpression negated the synergistic growth-inhibitory effect of IDF-11774 and bafilomycin A1 (Fig. 5f). Therefore, we examined whether bafilomycin A1 affects the interaction between Bcl-2 and Bax in the presence of IDF-11774 (Fig. 5g). A co-immunoprecipitation assay demonstrated that bafilomycin A1 abolished the interaction between Bcl-2 and Bax, resulting in the induction of apoptosis. Similarly, interaction of Bcl-xL with Bax was abrogated in the presence of IDF-11774 and bafilomycin A1 (Supplementary Figure 3C).

To confirm the significance of the Bcl-2 level in the synthetic lethal interaction of IDF-11774, we conducted isobologram analysis and determined the changes in the CI of IDF-11774 and bafilomycin A1 in WiDr cells overexpressing *BCL2* (Fig. 5h, Supplementary Figure 3D) and in *BCL2*-knockdown LoVo cells (Fig. 5h, Supplementary Figure 3E). As expected, overexpression of *BCL2* in WiDr cells increased the CI from 0.85 to 1.05, while *BCL2* knockdown in LoVo cells reduced the CI from 1.16 to 0.98, confirming the significant effect of the Bcl-2 level on the synthetic lethality of IDF-11774 and bafilomycin A1. The correlation between Bcl-2 expression and the CIs of IDF-11774 with inhibitors of autophagosome–lysosome fusion suggested that Bcl-2 plays a crucial role in the synthetic lethal interaction of IDF-11774 with ATP6V0C.

On the basis of our findings, we present a model of the Bcl-2-dependent synthetic lethal interaction of IDF-11774 and ATP6V0C, as shown in Fig. 5i. Suppression of autolysosome formation by *ATP6V0C* knockdown or bafilomycin A1 induces a switch from autophagy formation to apoptosis induction, which results in the release of Bcl-2/Bcl-xL from the Bcl-2-Bax/Bak complex.

### Bcl-2-dependent growth inhibition of conditionally reprogramed cells (CRCs) by combined treatment with IDF-11774 and autophagy inhibitors

**Fig. 6:**
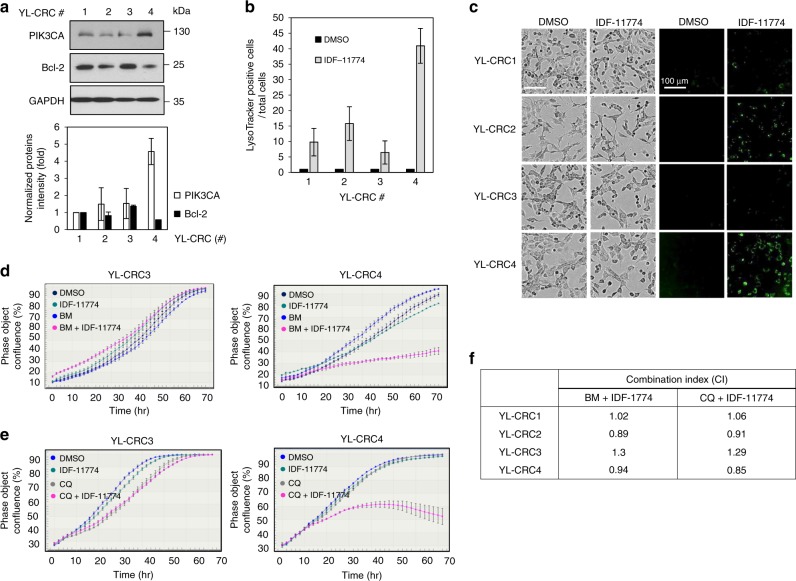
Regulation of IDF-1174 sensitivity to conditionally reprogramed cells (CRCs) derived from patients with colon cancer. **a** Western blot analysis of Bcl-2 expression in the CRCs. The signal intensity of each protein was normalized to that of GAPDH. **b**, **c** Autolysosome formation in CRCs treated with 10 µM IDF-11774. Scale bar, 200 µm. Bars indicate the ratio of the average of LysoTracker-positive cells in different regions (*n* = 9) of the well. **d**, **e** Growth inhibition of CRCs expressing different levels of Bcl-2 in the presence of 1 nM bafilomycin A1 and 5 µM IDF-11774 or 5 µM chloroquine, as measured using IncuCyte ZOOM system. Data are presented as the single representative result of three experiments and as the mean ± S.D. In each experiment, each treatment was repeated in triplet wells, and each well was analyzed in 4 frames. **f** Combination index of IDF-11774 with bafilomycin A1 (BM) or chloroquine (CQ) in CRCs

To evaluate the potential clinical application of IDF-11774 in patients with colorectal cancer, we examined the synthetic lethality of IDF-11774 with ATP6V0C using bafilomycin A1 or chloroquine in patient-derived YL-CRCs. Western blot analysis demonstrated low levels of Bcl-2 in YL-CRC2 and YL-CRC4 (Fig. 6a).

We then investigated autolysosome formation in YL-CRCs in the presence of IDF-11774. IDF-11774-induced LysoTracker fluorescence was stronger in both YL-CRC2 and YL-CRC4 than in YL-CRC1 and YL-CRC3 (containing high levels of Bcl-2) (Fig. 6b, c). Combined treatment with IDF-11774 and bafilomycin A1 or chloroquine inhibited the growth of YL-CRC4, but not YL-CRC3 (Fig. 6d, e). Isobologram analysis revealed a synergistic effect of IDF-11774 with bafilomycin A1 or chloroquine in YL-CRC2 and YL-CRC4, and an antagonistic effect in YL-CRC1 and YL-CRC3 (Fig. 6f, Supplementary Figure 4). These results suggested that Bcl-2 expression is a crucial biomarker for the clinical application of synthetic lethality of IDF-11774 with ATP6V0C.

## Discussion

Stimuli such as rapamycin treatment, starvation, and an increase in the cellular AMP/ATP ratio activate AMPK to repress mTOR, which directly phosphorylates ULK1 and initiates autophagy.^32,33^ Autophagy plays an important role in cell survival, development, and cancer progression.^34^ However, autophagy can serve as a back-up mechanism when apoptosis is defective, and can lead to type II programmed cell death in collaboration with apoptosis.^33,35^ Many anti-cancer drugs induce autophagy, thereby conferring chemoresistance, which is relieved by V-ATPase inhibitors in cancer cells.^36–38^ However, the mechanism of sensitization of cancer cells to anti-cancer drugs by V-ATPase inhibitors has remained unclear.^37,39^ Here we report that the novel HSP70 inhibitor, IDF-11774, showed a Bcl-2-dependent synthetic lethal interaction with an ATP6V0C inhibitor, resulting in synergistic apoptosis of colorectal cancer cells.

Chemogenetic RNAi screening to identify synthetic lethal interactions is useful to establish therapeutic strategies for precision cancer medicine.^7^ In our genome-wide RNAi screen, genes involved in cell cycle, proteolysis, and the lysosome were identified as candidates of synthetic lethality with IDF-11774. For further analysis, we selected *ATP6V0C*, which encodes a critical component of an ATP-driven proton pump V-ATPase and regulates functional autolysosome formation and the acidic tumor microenvironment.^27,40^ Genetic depletion of *ATP6V0C* disrupted lysosomal acidity and blocked autophagic flux, thereby generating non-functional autolysosomes.^41^ V-ATPase is upregulated in hepatocellular carcinoma^42^ and knockdown of *ATP6L* blocked the metastasis of hepatocellular carcinoma in a xenograft model.^43^

Our study revealed that *ATP6V0C* knockdown blocked IDF-11774-induced functional autolysosome formation and enhanced the sensitivity of cancer cells to IDF-11774, indicating that their synthetic lethal interaction. Moreover, the synergistic effect of IDF-11774 treatment and *ATP6V0C* knockdown correlated with the extent of IDF-11774-mediated functional autolysosome formation in various colorectal cancer cell lines. Combined treatment with IDF-11774 and bafilomycin A1, an ATP6V0C inhibitor, resulted in synergistic apoptosis by blocking autolysosome formation in HCT116 and WiDr cells. Moreover, combination of IDF-11774 and Bafilomycin A1 gave significant inhibition of tumor growth in mouse model of HCT116 (Supplementary Figure 5). Similarly, other inhibitors of autolysosome formation, such as chloroquine and concanamycin A, caused substantial inhibition of cancer cell growth when combined with IDF-11774. Contrastingly, proton-pump inhibitors, such as omeprazole, pantoprazole, and lansoprazole had no effect on sensitivity to IDF-11774 (Supplementary Figure 6).

*PIK3CA* mutations, which confer metastatic properties to colorectal cancer cells, are present in HCT116, WiDr, and HT29, but not in LoVo, colo320, and SW620 cells. A recent study demonstrated that cell lines bearing the H1047R mutation in PIK3CA downregulates Bcl-2, thereby promoting tumorigenesis and migration.^44^ Bcl-2 suppresses not only autophagy formation by binding to Beclin 1/Atg6, but also apoptosis by blocking the pro-apoptotic activities of Bax and Bak.^35,45^ Our data revealed that autolysosomes were easily formed in *BCL2*-knockdown cells, but not in *BCL2*-overexpressing cells in the presence of IDF-11774. Moreover, binding of Bcl-2 to Bax was abrogated in the presence of IDF-11774 and bafilomycin A1, leading to apoptosis in cancer cells. Notably, the synthetic lethal interaction of IDF-11774 with *ATP6V0C* was observed in HCT116 and WiDr cells with low Bcl-2 levels, but not in LoVo or colo320 cells with high Bcl-2 levels. In contrast, the growth of CCD-18Co cells, which are normal colonocytes with relatively low levels of Bcl-2, was not affected by treatment with IDF-11774 and bafilomycin A1 (Supplementary Figure 7). As expected, *BCL2* knockdown reduced the CI of simultaneous treatment of IDF-11774 and bafilomycin A1, while *BCL2* overexpression increased the CI. Likewise, YL-CRC2 and YL-CRC4, with low levels of Bcl-2, exhibited more autolysosome formation and stronger synergistic growth inhibition upon combined treatment with IDF-11774 and bafilomycin A1 or chloroquine compared with YL-CRC1 and YL-CRC3, which have high levels of Bcl-2. Moreover, anti-apoptotic protein Bcl-xl (*BCL2L1*), which was identified as a candidate for synthetic lethality with IDF-11774, showed similar effects to Bcl-2 (Fig. 1a, Supplementary Figure 8A). However, the pro-apoptotic Bak1 did not affect the synergistic effects of IDF-11774 and bafilomycin A1 (Supplementary Figure 8B).

Although our chemogenetic RNAi screening successfully identified synthetic lethal genes for IDF-11774, it has some technical limitations. We cannot exclude the possibility of false negatives because of the variability in the knockdown efficacy of more than 27,500 shRNAs of the 5043 genes included in the screening, and false positives caused by off-target silencing effects. Moreover, we might have excluded shRNAs for potential synthetic lethal genes in the screening. Recent developments in RNAi and CRISPR/Cas9 technologies and careful selection of the shRNA library might improve the identification of synthetic lethal genes in future chemogenetic RNAi screenings.

The findings of the present study are important in the light of clinical application and directions for future research. First, we identified a synthetic lethal interaction of IDF-11774 with *ATP6V0C*, resulting in synergistic apoptosis of colorectal cancer cells. Second, we elucidated the mechanism of chemosensitization of cancer cells to IDF-11774 by ATP6V0C inhibitors via autolysosome formation. Finally, we identified that the Bcl-2 level is a crucial and clinically applicable biomarker for combinatorial treatment using IDF-11774 and ATP6V0C inhibitors.

## Electronic supplementary material

Supplementary Figure 1. Evaluation of candidate target genes

Supplementary Figure 2. Immunohistochemistry of ATP6V0C in colorectal cancer tissue arrays

Supplementary Figure 3. Bcl-2 expression level is crucial for the synthetic lethality of IDF-11774 and bafilomycin A1

Supplementary Figure 4. Isobologram analysis

Supplementary Figure 5. In-vivo combined therapy of IDF-11774 and bafilomycin A1 in an HCT116 xenograft model

Supplementary Figure 6. Combinatorial growth inhibitory effect of IDF-11774 and proton-pump inhibitors in HCT116 cells. Combinatorial effect of IDF-11774 and bafilomycin A1

Supplementary Figure 7. Effect of a combination treatment with Bafilomycin A1 and IDF‑11774 on the growth of normal colon CCD-18Co cells

Supplementary Figure 8. Effect of Bcl-2 family proteins on synergistic interaction of IDF-11774 and Bafilomycin A1

Supplementary Table 1. Sequences of the siRNA

Supplementary Table 2. Sequences of the primers

Supplementary Table 3. Data points of SRB assay in Fig. 3b
