# Supplementary figures and images for: Bcl-2-dependent synthetic lethal interaction of the IDF-11774 with the V0 subunit C of vacuolar ATPase (ATP6V0C) in colorectal cancer
Source: Br J Cancer. 2018 Nov 13;119(11):1347–57. doi: 10.1038/s41416-018-0289-1 (PMC6265273; doi:10.1038/s41416-018-0289-1)

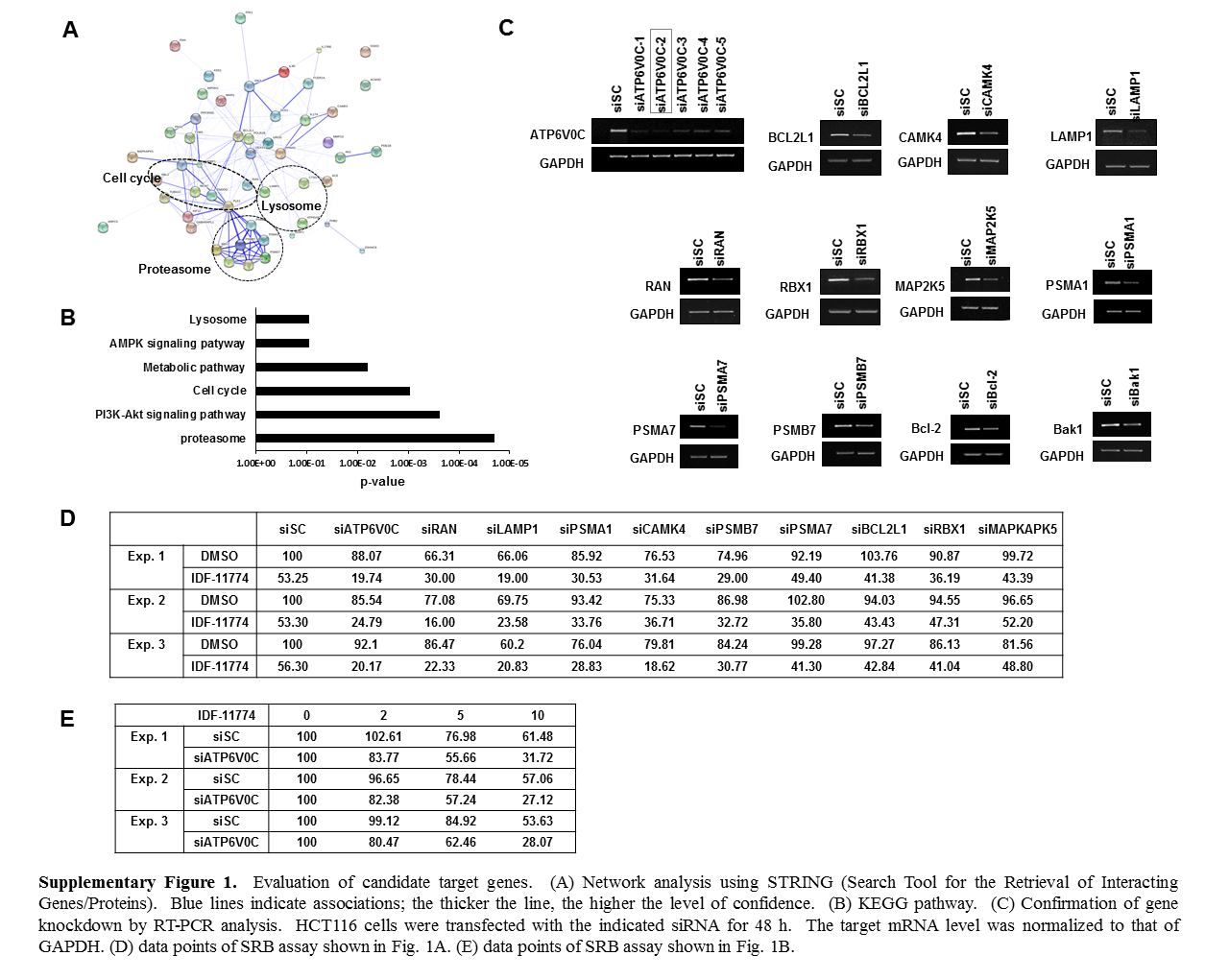

Supplement: Supplementary file 1 — Supplementary Figure 1. Evaluation of candidate target genes [file 41416_2018_289_MOESM1_ESM.tif]

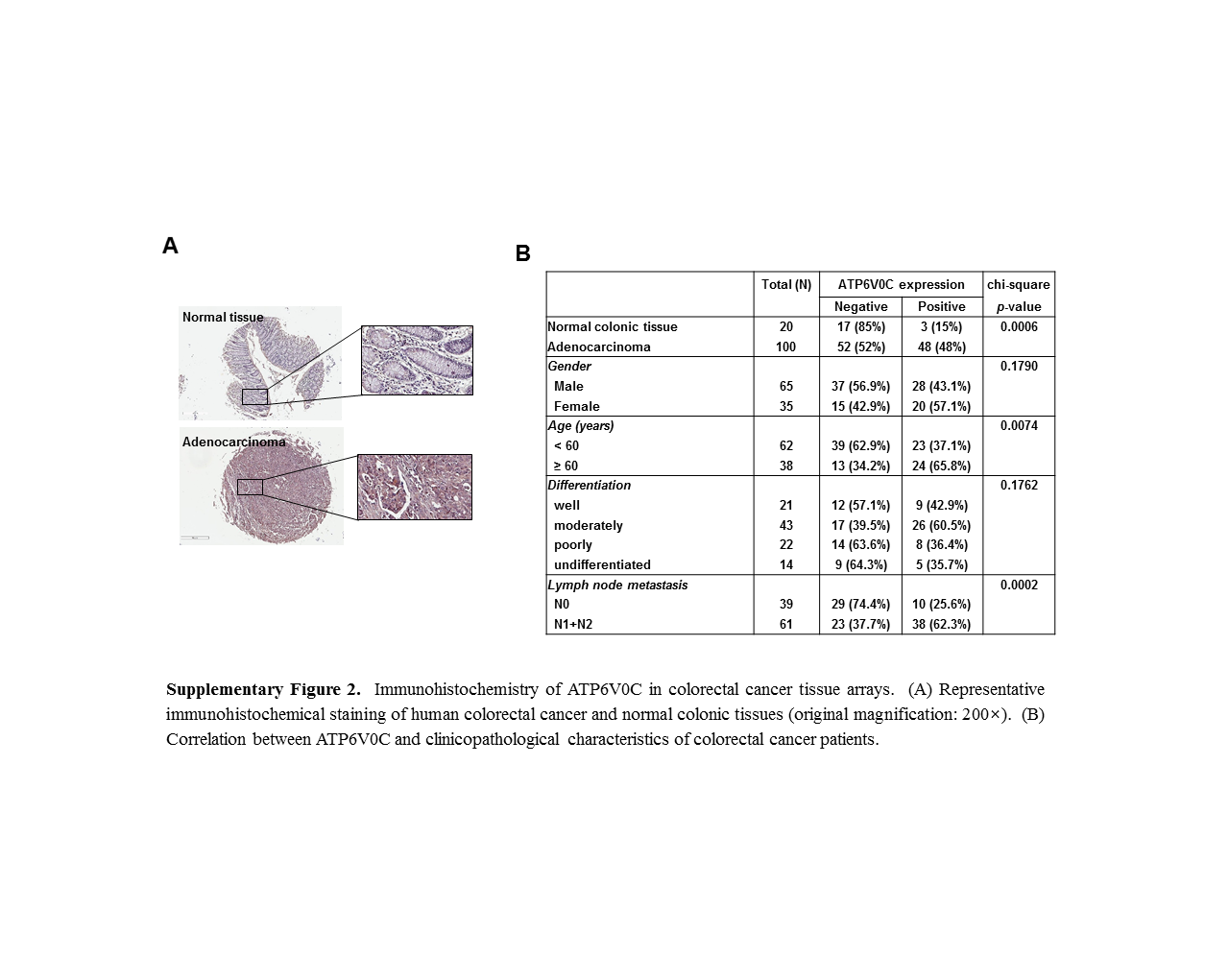

Supplement: Supplementary file 2 — Supplementary Figure 2. Immunohistochemistry of ATP6V0C in colorectal cancer tissue arrays [file 41416_2018_289_MOESM2_ESM.tif]

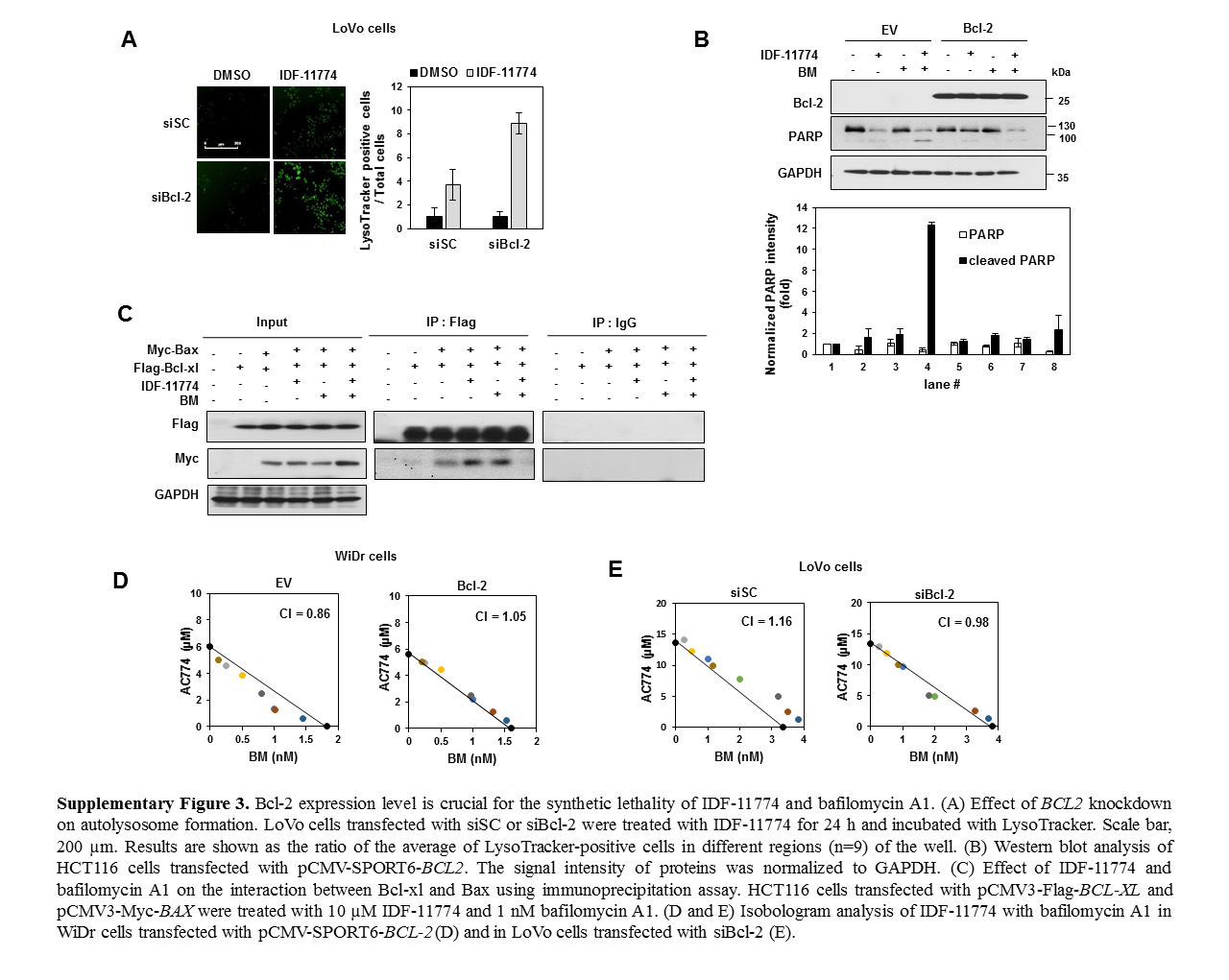

Supplement: Supplementary file 3 — Supplementary Figure 3. Bcl-2 expression level is crucial for the synthetic lethality of IDF-11774 and bafilomycin A1 [file 41416_2018_289_MOESM3_ESM.tif]

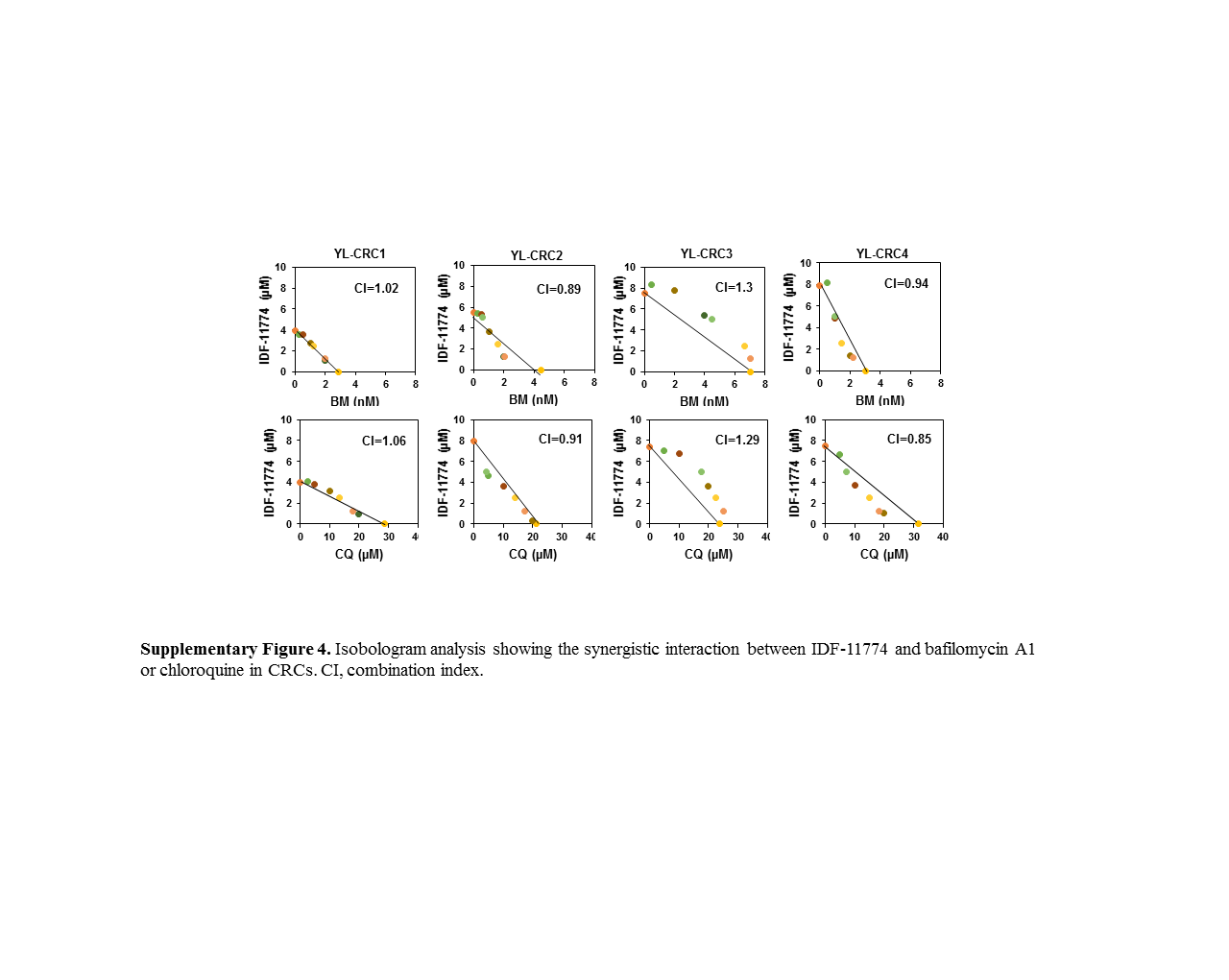

Supplement: Supplementary file 4 — Supplementary Figure 4. Isobologram analysis [file 41416_2018_289_MOESM4_ESM.tif]

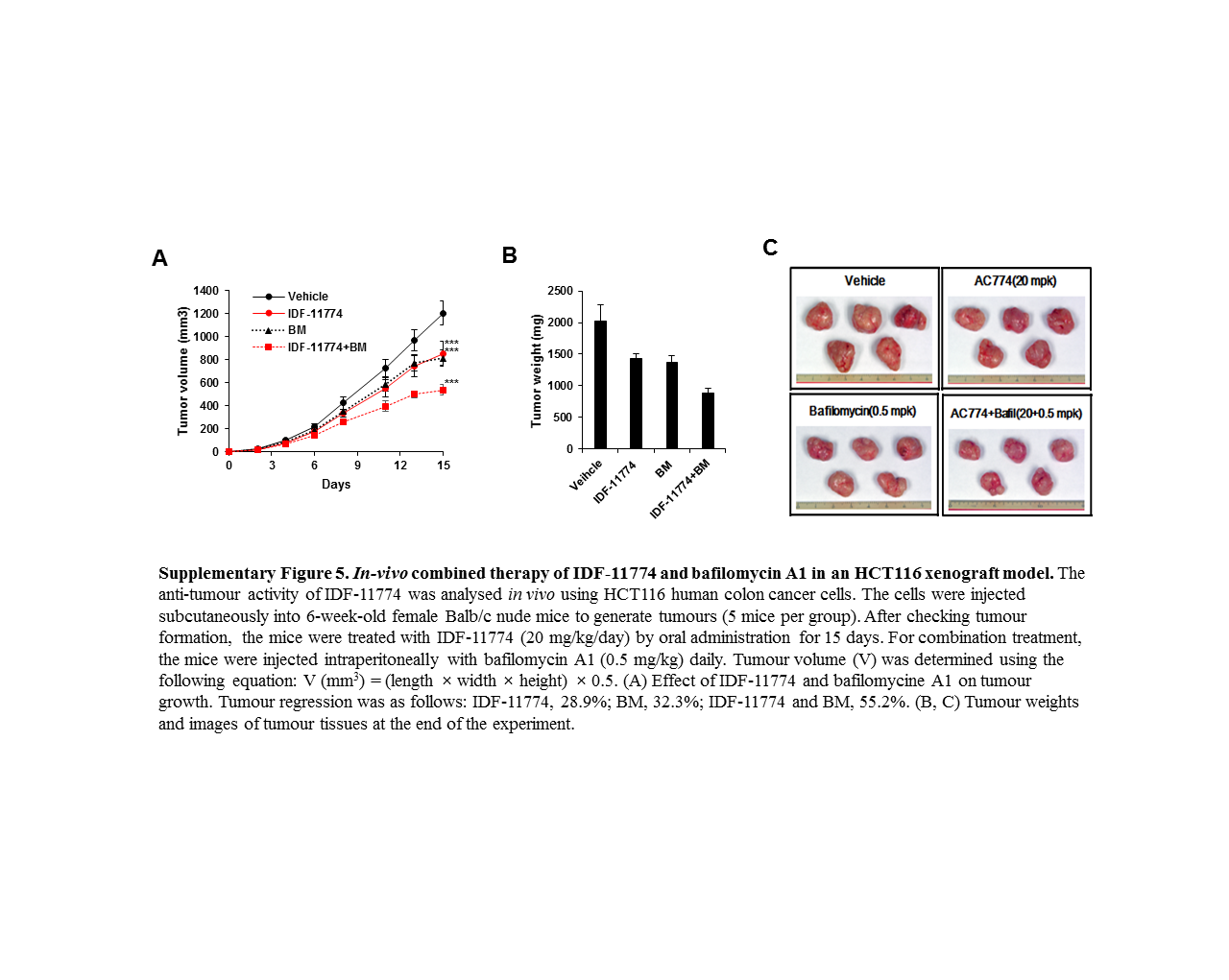

Supplement: Supplementary file 5 — Supplementary Figure 5. In-vivo combined therapy of IDF-11774 and bafilomycin A1 in an HCT116 xenograft model [file 41416_2018_289_MOESM5_ESM.tif]

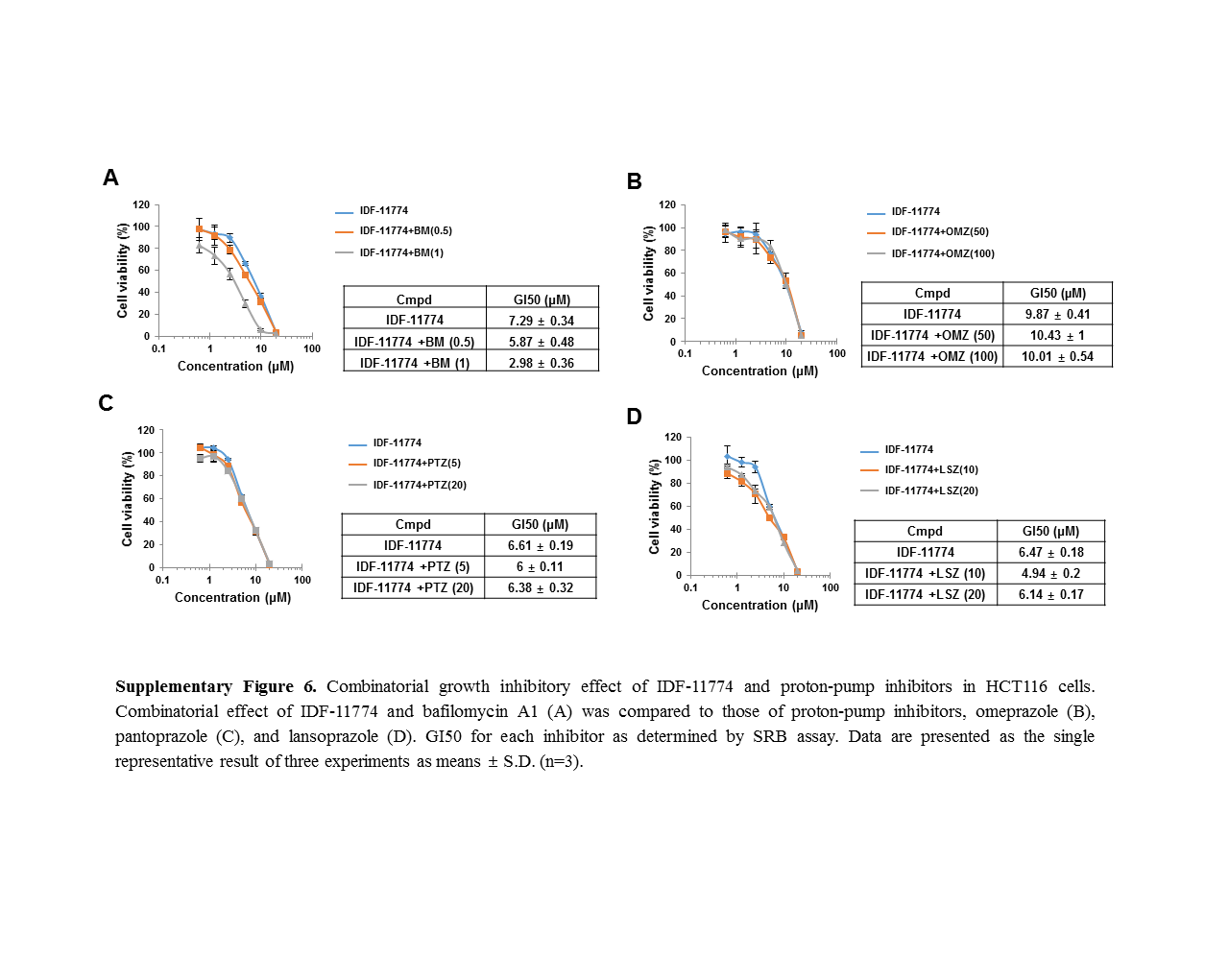

Supplement: Supplementary file 6 — Supplementary Figure 6. Combinatorial growth inhibitory effect of IDF-11774 and proton-pump inhibitors in HCT116 cells. Combinatorial effect of IDF-11774 and bafilomycin A1 [file 41416_2018_289_MOESM6_ESM.tif]

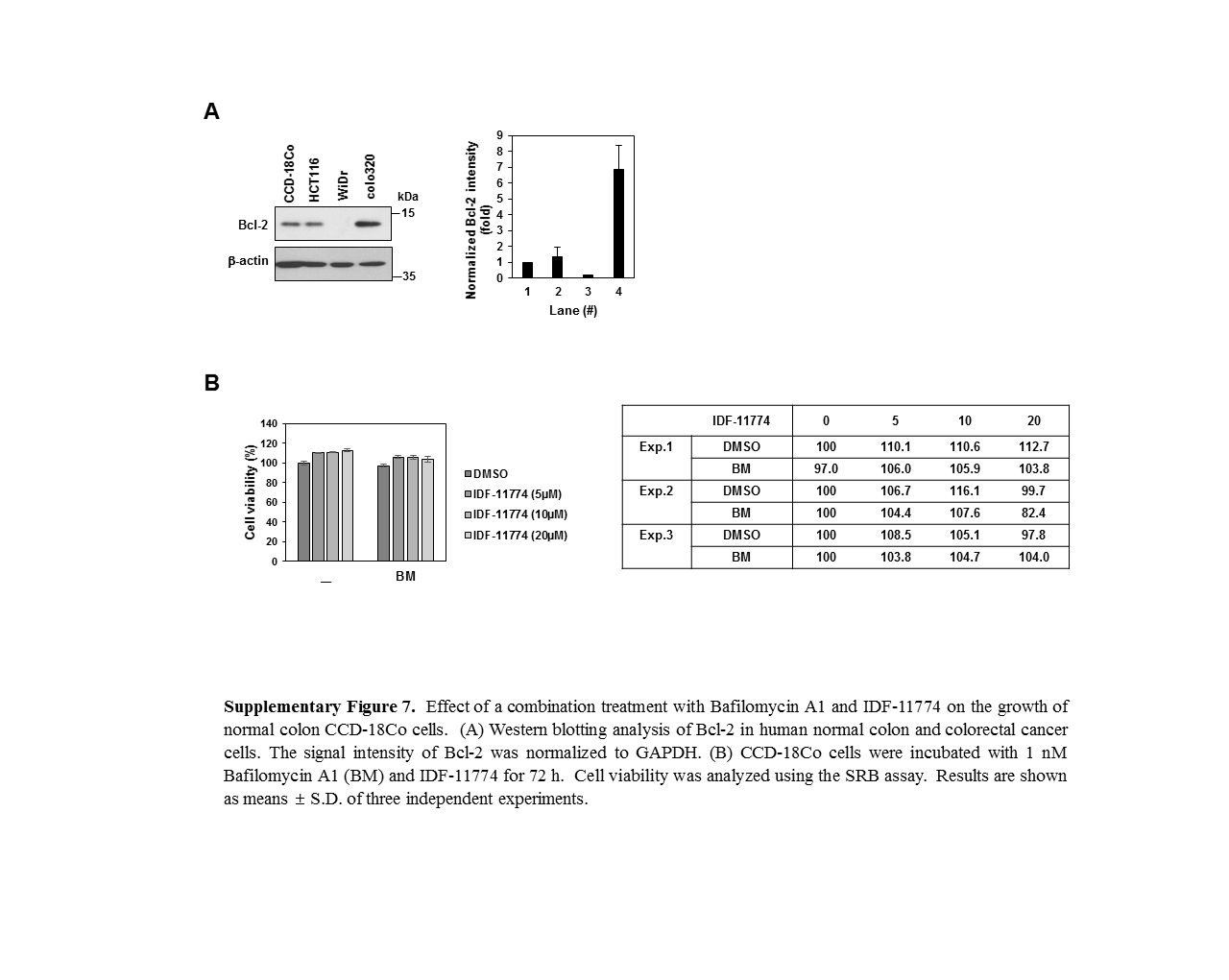

Supplement: Supplementary file 7 — Supplementary Figure 7. Effect of a combination treatment with Bafilomycin A1 and IDF‑11774 on the growth of normal colon CCD-18Co cells [file 41416_2018_289_MOESM7_ESM.tif]

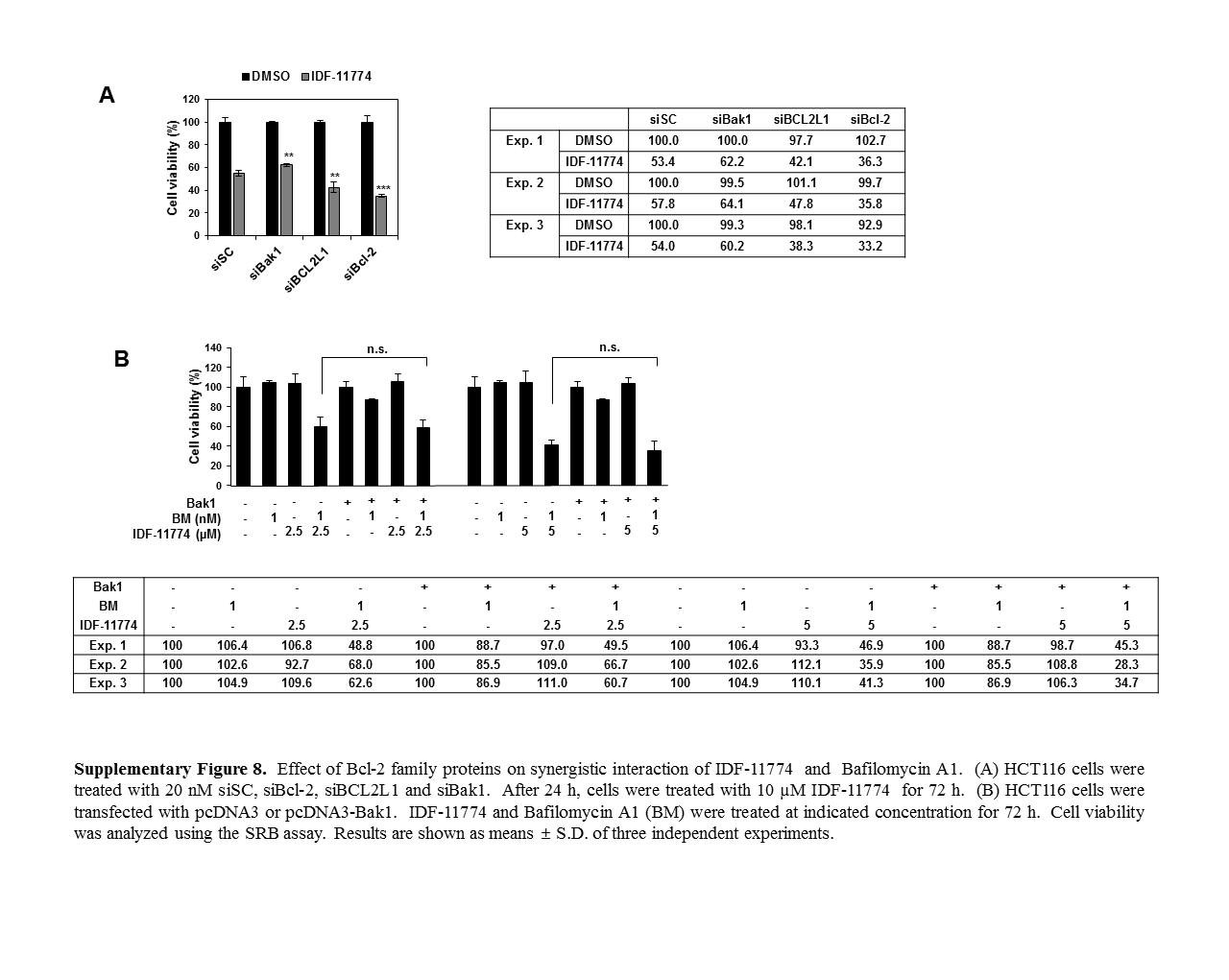

Supplement: Supplementary file 8 — Supplementary Figure 8. Effect of Bcl-2 family proteins on synergistic interaction of IDF-11774 and Bafilomycin A1 [file 41416_2018_289_MOESM8_ESM.tif]

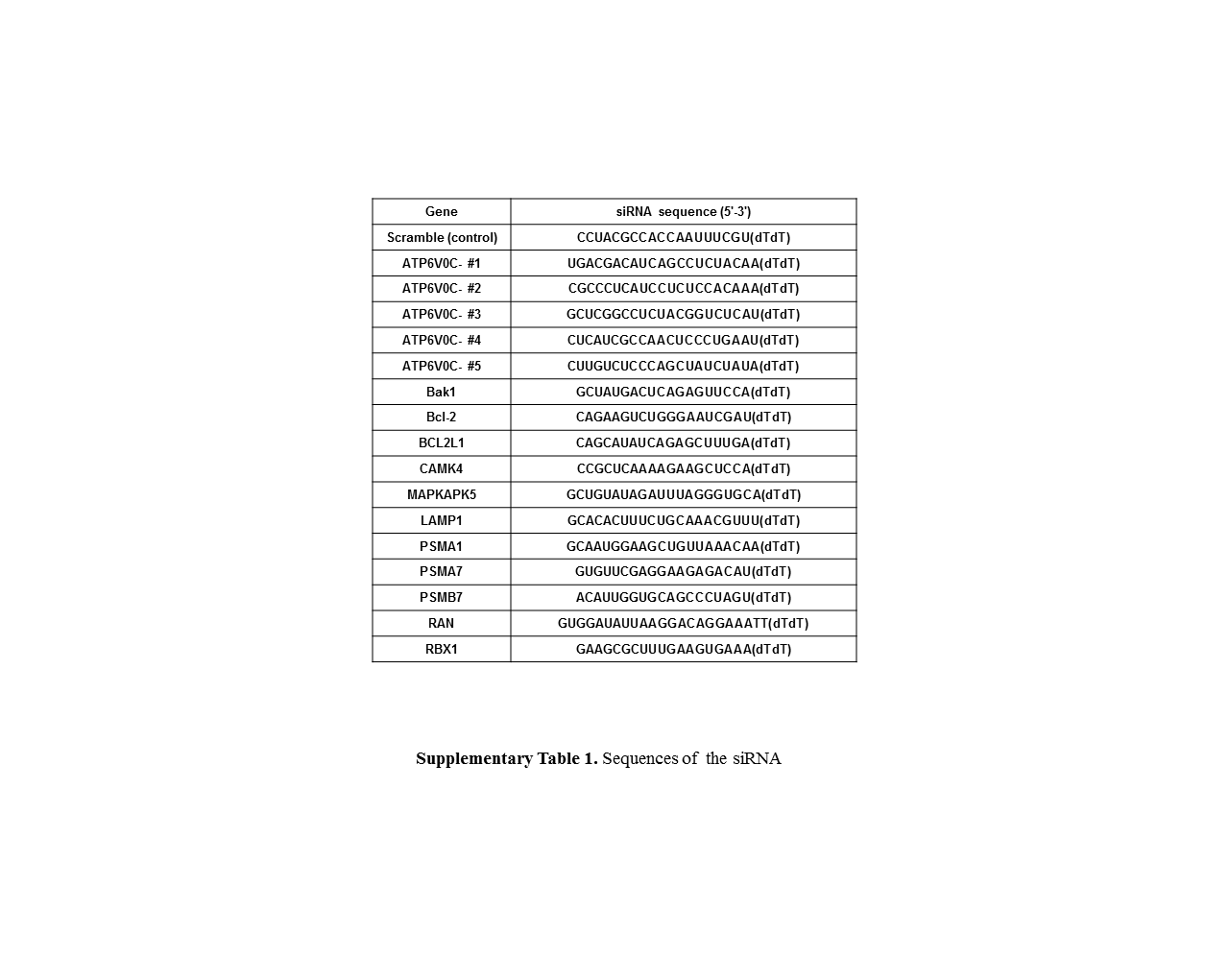

Supplement: Supplementary file 9 — Supplementary Table 1. Sequences of the siRNA [file 41416_2018_289_MOESM9_ESM.tif]

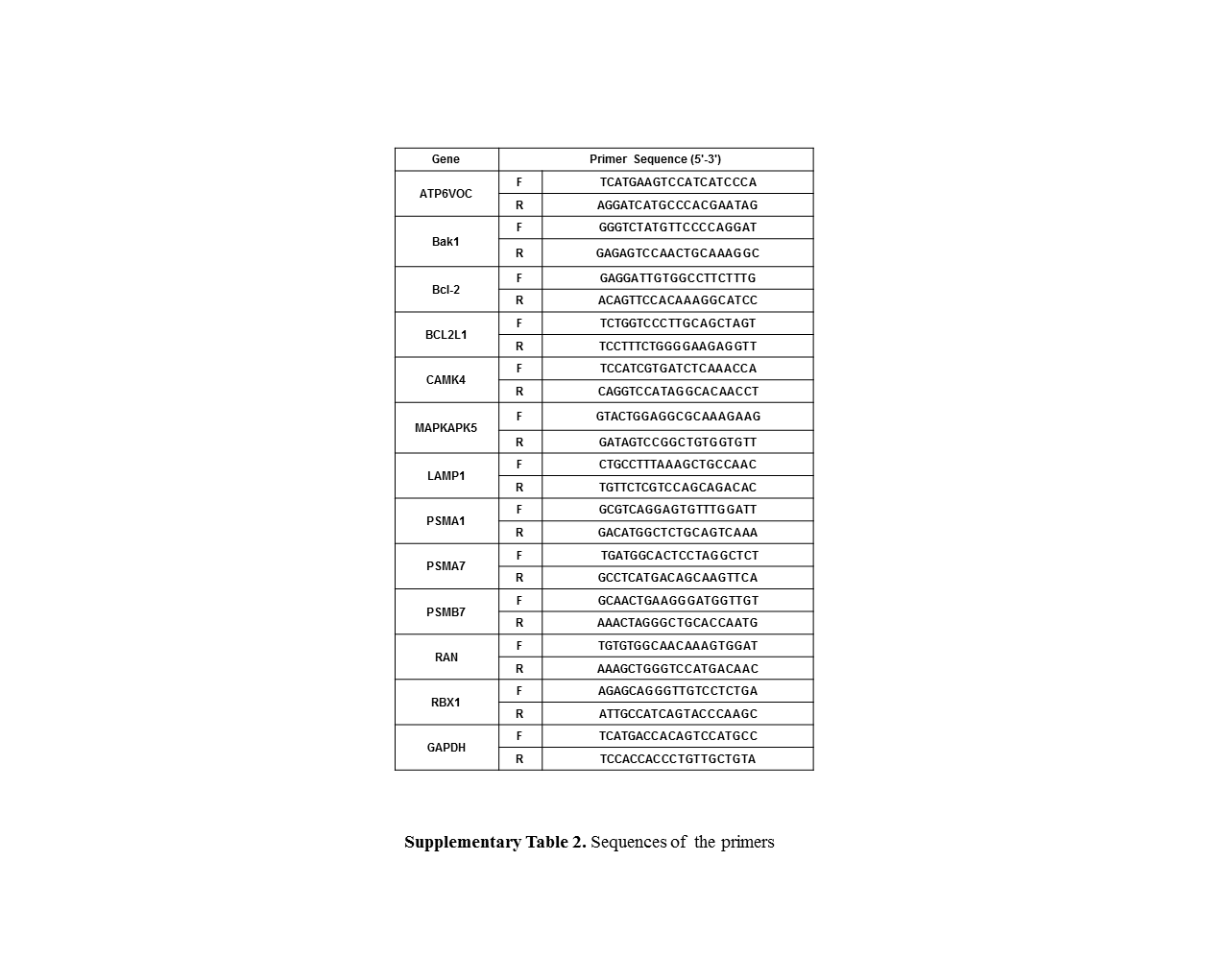

Supplement: Supplementary file 10 — Supplementary Table 2. Sequences of the primers [file 41416_2018_289_MOESM10_ESM.tif]

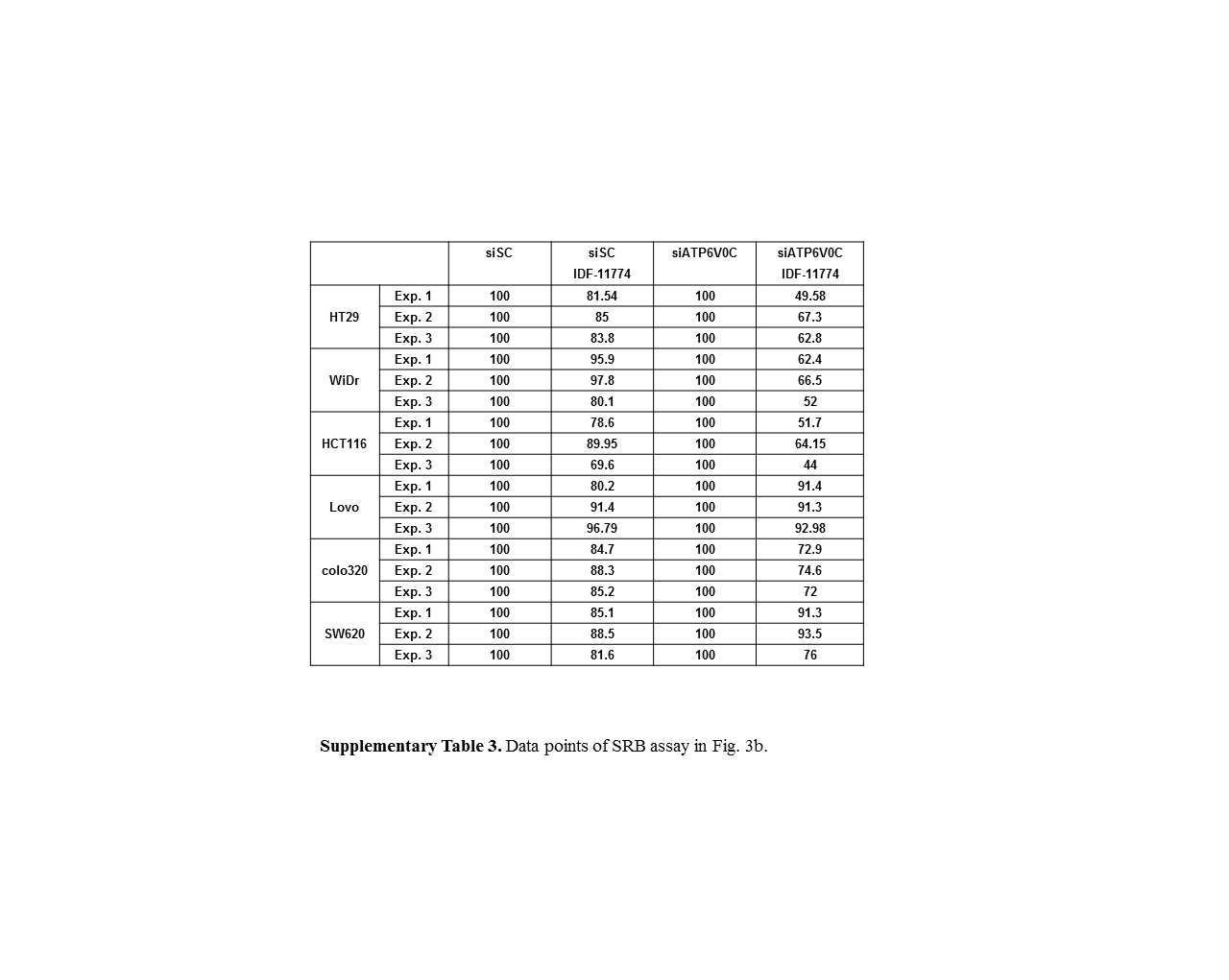

Supplement: Supplementary file 11 — Supplementary Table 3. Data points of SRB assay in Fig. 3b [file 41416_2018_289_MOESM11_ESM.tif]
